# 

**Published:** 1895-04

**Authors:** 


					﻿vol. XV.
APRIL, 1895.
No. 4.
THE
]1OM(EOPATH1C PHYSICIAN,
A Monthly Journal of Homoeopathic Materia Medica
and Clinical Medicine.
“ He is a freeman whom truth makes free, and all are slaves beside."
“Seek the Truth: come whence it may, cost uhal it will.”
BDITBD AND PUBLISHED BY
WALTER M. JAMES, M. D.
PHILADELPHIA :
JSTo. 1231 LOCUST STREET.
London:-
ALFRED HEATH & CO., Sole Agents bob Gbeat Bbitain,
114 Ebury Street, Eaton Square.
<J*FearZ?/ Subscription, $2.SO, invariably in advance. Single numbers, 2S els.
Entered at the Philadelphia PeevOfflee an Seoeod-Claea Mail Mather.
				

